# Altered resting‐state hippocampal functional connectivity in breast cancer survivors with chemotherapy‐induced amenorrhea

**DOI:** 10.1002/brb3.3039

**Published:** 2023-05-08

**Authors:** Yingying Zhuang, Lili Guo, Wei Huang, Genji Bo, Jiandong Zhang, Zhaohuan Zhu, Yun Feng

**Affiliations:** ^1^ Department of Medical Imaging Medical Imaging Center, The Affiliated Huaian No. 1 People's Hospital of Nanjing Medical University Huai'an Jiangsu China

**Keywords:** breast cancer, chemotherapy, chemotherapy‐induced amenorrhea, functional connectivity, hippocampus

## Abstract

**Introduction:**

Amenorrhea induced decrease of hormones is associated with cognitive impairment. This study aimed to evaluate hippocampal functional connectivity patterns in chemotherapy‐induced amenorrhea (CIA) breast cancer (BC) patients, to evaluate the relationship between the functional connectivity features and hormone levels.

**Method:**

Neuropsychological test, functional magnetic resonance imaging, and assessment of hormone levels were conducted in 21 premenopausal BC patients before chemotherapy (*t*_0_) and 1 week after completing chemotherapy (*t*_1_). Twenty matched healthy controls (HC) were also included and underwent the same assessments at similar time intervals. Mixed effect analysis and paired *t*‐test were used to compare differences in brain functional connectivity.

**Results:**

Voxel‐based paired *t*‐tests revealed increased functional connectivity of the right and left hippocampus with the left fusiform gyrus, inferior and middle temporal gyrus, inferior occipital gyrus, left lingual gyrus, and parahippocampal gyrus after chemotherapy (*p* < .001) in CIA patients. Repeated measures analysis revealed significant group‐by‐time interactions in the left hippocampus with the bilateral fusiform gyrus, right parahippocampal gyrus, left inferior temporal gyrus, and left inferior occipital gyrus (*p* < .001). Premenopausal BC patients had no significant differences in cognitive function compared with HC at baseline. However, the CIA patients had high levels of self‐rating depression scale, self‐rating anxiety scale, total cholesterol, and triglycerides. Further, the CIA patients showed significant differences in hormone and fasting plasma glucose levels and cognitive performances between *t*_0_ and *t*_1_ (*p* < .05). Functional connectivity changes between the left hippocampus and the left inferior occipital gyrus was negatively correlated with E2 and luteinizing hormone changes (*p* < .05).

**Conclusion:**

The CIA patients had cognitive dysfunction mainly in memory and visual mobility. Chemotherapy may affect hippocampal‐posterior cortical circuit which mediates visual processing in CIA patients. Moreover, E2 may be involved in this process.

## Graphical abstract

This study revealed that CIA induced abnormal brain activity may overlap with the mechanism of chemotherapy‐induced cognitive impairment, which suggesting a possible compound effect. The regulation of neuroplasticity by E2 provides a framework for predicting estrogen‐dependent cognitive function in the brain.

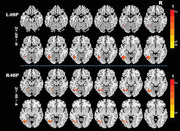

## 1 INTRODUCTION

Chemotherapy‐induced amenorrhea (CIA) is a potential long‐term side effect of breast cancer (BC) survivors. Amenorrhea occurs in about 20%–80% of premenopausal BC patients receiving adjuvant chemotherapy (Torino et al., 2014; Zhang et al., 2018). Amenorrhea may be associated with considerable morbidity or discomfort symptoms. In addition, amenorrhea induced hormone disorders may be associated with cognitive impairment and negative emotions (Vearncombe et al., 2009). Schagen et al. (1999) reported decreased cognitive performance in multiple domains of the CIA group compared to non‐CIA group, suggesting that CIA was associated with increased chemotherapy‐induced cognitive impairment (CRCI). Both CIA and surgical menopause may cause a sudden decline of ovarian hormones. Consequently, this may induce menopause and a decline in cognitive function. Sherwin et al. reported that surgical menopause was associated with deleterious effects on specific cognitive domains. Women who did not receive estrogen treatment after oophorectomy showed an immediate and delayed reduction in abstract reasoning and reaction speed in verbal memory (Sherwin & Phillips, 2006). Studies have shown that estradiol has important cognitive regulatory functions, such as promoting synaptic formation in mature brain areas (Vierk et al., 2015). In addition, estrogens have been shown to have neuroprotective effects (Luine & Frankfurt, 2020). Our previous study showed that altered hippocampus functional connectivity after chemotherapy was associated with decreased estrogen levels, suggesting that chemotherapy causes an abrupt decrease in estrogen and decreased neurocognitive performance (Feng et al., 2019).

The hippocampus is an important anatomical structure of the limbic system, which plays an important role in encoding and storing memory, and regulating stress and emotions. It is increasingly becoming the focus of current research due to its high vulnerability to direct (chemotherapy, radiotherapy, and endocrine therapy) and indirect (tumor‐related stress or negative emotions) treatment‐related side effects (Peukert et al., 2020). Estrogen effects in the brain are mediated through estrogen receptor subtypes, ERα and ERβ, expressed in the hippocampus (Carrier et al., 2015). A previous study showed that rats treated with estrogen showed increased density of dendritic spines in the hippocampal CA1 pyramidal cells (Brandt et al., 2020). Furthermore, early estrogen replacement in ovariectomized mice increased proliferation of hippocampal stem cells (Sha et al., 2015). In addition, estrogen can increase cerebral blood flow by promoting the release of endothelial relaxation factors, as patients on estrogen replacement therapy showed increased cerebral blood flow in the hippocampus and temporal lobes (Maki & Resnick, 2000). Therefore, we hypothesized that CIA causes abrupt hormone changes affecting hippocampal structure and function. However, few studies have used neuroimaging methods to investigate the cognitive effects of CIA.

Chemotherapy is associated with decreased hippocampal volume (Inagaki et al., 2007), structural distortions (Apple et al., 2017), and alterations in functional connectivity (Kesler & Blayney, 2016). The hippocampus is also highly sensitive to radiotherapy and endocrine therapy, which are also associated with decreased hippocampal volume and changes in functional connectivity (Chen et al., 2017; Guo et al., 2018). A few longitudinal studies have focused on neuroimaging of the hippocampal structure and functional connectivity changes with CIA in BC survivors (Conroy et al., 2013). However, the neural mechanism responsible for the changes is still unknown. A previous study showed that post‐chemotherapy hippocampal functional connectivity was correlated to changes in anxiety, E2, and triglyceride levels in BC patients (Feng et al., 2020). However, the study had a limitation in that it included older postmenopausal women. Thus, this prospective, longitudinal study aimed to determine the hormonal and metabolic influences on hippocampal‐whole brain functional connectivity during CIA.

## 2 MATERIALS AND METHODS

### 2.1 Participants

Written informed consent was obtained from all study participants. The study protocol was approved by the Nanjing Medical University Research Ethics Committee in accordance with the Declaration of Helsinki. A total of 21 premenopausal women with primary nonmetastatic BC (pre‐BC) (stages I–III) scheduled for adjuvant chemotherapy (age 44.0 ± 5.1 years) and 20 matched healthy controls (HC) (age 40.3 ± 6.5 years) were included in the study. All participants had attained at least a minimum of 9 years of education. The study participants underwent brain magnetic resonance imaging (MRI) scanning, neuropsychological testing, and blood biochemical assessments. Pretreatment assessment was done before the operation or neoadjuvant chemotherapy (*t*_0_, baseline), whereas follow‐up assessment was conducted 1 week after completing chemotherapy (*t*_1_, 5–6 months after *t*_0_). All participants had a regular menstrual cycle. Patients with neuropsychiatric disorders, brain tumors or injury, alcohol abuse or drug addiction, using psychotropic medication, and other chronic diseases affecting cognitive function assessment were excluded from the study.

Thirteen patients (13/21, 61.9%) received four cycles of ACT regimen (doxorubicin, cyclophosphamide, and paclitaxel intravenously), whereas eight patients (8/21, 38.1%) received six cycles of TEC regimen (docetaxel, epirubicin, and cyclophosphamide intravenously). The full regimen was administered over 5–6 months. Radiotherapy and endocrine therapy were not administered during the chemotherapy. The HC received no intervention. However, they were evaluated within 5–6 months of the baseline testing.

#### 2.1.1 Neuropsychological assessment

All participants were evaluated using a battery of neuropsychological tests to assess cognitive ability. The general cognitive function was assessed with the Beijing version of the mini‐mental state examination (MMSE), and MMSE > 26 score was identified as having normal cognition. Attention/executive function assessment (number connection test A); processing speed (digit symbol test); visual ability (line tracing test, LTT); the fine motor skills (serial dotting test, SDT); reaction capability (the Chinese version of the Stroop color‐word test); short‐term and long‐term memory (auditory verbal learning test, WDT). All the participants’ anxiety and depression symptoms were evaluated using self‐rating depression scale (SDS) and self‐rating anxiety scale (SAS), respectively. The neuropsychological tests would be conducted by the same skilled researcher in 60 min.

#### 2.1.2 Biochemical assessment

About 10 mL of fasting venous blood sample was collected from the forearm of the patients in the morning before the MRI scan. The blood was stored at room temperature and was sent for laboratory analysis within 2 h of collection. The blood tests included fasting blood glucose concentration, hemoglobin (Hb) concentration, triglyceride levels, total cholesterol, and hormone levels.

The blood samples were collected between four to 10 days after the end of menstruation. The blood was centrifuged at 3000 r/min for 20 min to obtain serum. Chemiluminescent immunoassay was used for quantification of the estradiol (E2), luteinizing hormone (LH), and follicular stimulating hormones (FSH).

### 2.2 MRI data acquisition

All the participants’ functional and structural images were collected by the same 32‐channel 3.0‐T MRI scanner (Discovery MR750, GE HealthCare, Milwaukee, WI, USA). During MRI scans, all participants kept their eyes closed, relaxed, motionless, awake, and unintentional thinking condition. MRI scan paradigms had been described in our previous study in detail Feng et al., 2020. The high‐resolution 3D T1‐weighted brain volume images for anatomic reference were collected for accurate brain structure registration: slice thickness = 1.0 mm, repetition time (TR) = 8.14 ms, echo time (TE) = 3.17 ms, flip angle (FA) = 12°, matrix = 256 × 256, and number of slices = 176. The Rs‐MRI images data were acquired across 250 scans with a gradient echo EPI sequence (TR = 2000 ms, TE = 30 ms, FA = 90°, matrix size: 64 × 64). The resting state scan took 500 s. Routine T2‐fluid attenuated inversion recovery sequence was acquired to exclude primary brain pathology. The HC group completed the MRI examinations at the baseline and the matched time interval.

### 2.3 Image preprocessing

The functional MRI images preprocessing were completed using the Date Processing Assistant Advanced Version for Resting State fMRI Data (http://rfmri.org/DPASF). After discarding the first 10 time points for the magnetic signal stabilization, the remaining functional images of 240 time points were analyzed for the following processing: slice timing and head motion correction. Only participates’ images with head motion <1.5 mm translation or rotation <1.5° would be included, then 3D‐T1 images were co‐registered to the functional MRI images. The structural images were segmented into gray matter, white matter, and cerebrospinal fluid and then spatially normalized to the Montreal Neurological Institute (MNI) space (voxel size resampled to 3 × 3 × 3 mm^3^). The final functional images of normalization matrix were acquired and smoothed using a 6 × 6 × 6 mm^3^ isotropic Gaussian kernel at full‐width at half‐maximum. Finally, a linear regression analysis was used to remove the sources of spurious covariance, including the signals of six head motion, cerebrospinal fluid, and white matter.

### 2.4 Functional connectivity analyses

Functional neuroimage software tool—Rest (http://www.fil.ion.ucl.ac.uk/spm) was used for the analysis of Rs‐fMRI data. The left and right hippocampi were chosen as the regions of interest (ROIs) as previously published methods (Chen & Etkin, 2013; Feng et al., 2020). Then, the mean time series of the left and right hippocampus were determined. Further, Pearson correlation coefficients (*r*) were computed between the average time series of each ROI and the whole brain voxel time series. In addition, connectivity correlation coefficients were transformed to *Z* values after the Fisher *r*‐to‐*z* transformation. Finally, functional connectivity maps between the hippocampus and the whole brain were obtained. Based on the functional connectivity analysis results, the peak MNI coordinate with a spherical radius of 6 mm was defined as a seed, time series were then extracted from these 6 mm seeds using REST software and further correlation analysis were performed.

### 2.5 Statistical analyses

#### 2.5.1 Demographic and neuropsychological data

SPSS17.0 software was used to analyze the demographic and neuropsychiatric data (SPSS Inc., Chicago, IL, USA). The Kolmogorov–Smirnov test was used to determine the normality of the data. Normally distributed continuous variables were expressed as mean ± standard deviation ($\bar{x}$ ± *S*). An independent sample *t*‐test was used to determine between groups differences of the baseline values. Repeated measures ANOVA were used to analyze group‐by‐time interactions after controlling for age. Paired sample *t*‐test was used to measure the differences between time points within each group.

The difference in functional connectivity between the hippocampus and the whole brain was determined using the DPABI toolbox (http://rfmri.org/dpabi). The voxel‐based two‐sample *t*‐test, paired *t*‐tests, and repeated measures ANOVA were used to compare differences in connection parameters between the two groups at baseline, between the time points within each group, and group‐by‐time interactions, respectively. Bilateral hippocampal volume, SAS, and SDS were used as covariates to reduce potential effects on functional connectivity. The results were corrected by the Gaussian random field theory correction (GRF) (voxel *p*‐value <.001, cluster *p*‐value <.05) for multiple comparisons. The *Z* values of brain regions with the significant group‐by‐time interaction (all clusters survived the paired *t‐*test) were extracted. Correlations analyses of changes in the neuropsychological tests or clinical data with *Z* values changes were conducted in BC patients (calculated as *Δ* value = *t*_1_ − *t*_0_). A *p*‐value <.05 was considered statistically significant.

### 2.6 Results

A total of 48 participants were initially recruited for the study. However, two HCs and one BC patient did not complete the follow‐up assessment. On the other hand, one HC and one BC patient were excluded for excessive head movement. In addition, one patient with cerebellar tumor and one with pancreatitis after chemotherapy were excluded. Therefore, the final study sample size was 21 BC patients and 20 HC.

#### 2.6.1 Demographic and clinical data

The baseline demographics and clinical characteristics are shown in Table 1. In this study, CIA was defined as the cessation of menses for at least 3 consecutive months during chemotherapy in premenopausal patients. All patients were followed up by telephone or outpatient visits 6 months after completing chemotherapy. Seventeen (81.0%) premenopausal patients received postoperative chemotherapy, whereas four (19.0%) received neoadjuvant chemotherapy. Of the 21 patients with BC, 19 (90.5%) had amenorrhea after chemotherapy, whereas 2 (9.5%) had menstrual disorders. There was no significant difference in age, sampling time, education level, general cognition, and baseline hormone levels between the BC and HC groups (*p* > .05). However, there were significant differences in SAS, SDS, HB, total cholesterol, and triglyceride levels at *t*_0_ between the BC and HC groups (all *p* < .05).

**TABLE 1:** Participants demographic and clinical characteristics at baseline.

	Patients received chemotherapy (*n* = 21)	Healthy controls (*n* = 20)	*t*‐Test
	Mean ± SD or count	Mean ± SD	*p*‐Value
Age (years)	44.0 ± 5.1	40.3 ± 6.5	.051^b^
Education levels (years)	13.6 ± 3.4	14.9 ± 2.1	.060
Breast cancer stage *n* (%)			
I	4 (19.05%)	NA	NA
II	9 (42.85%)	NA	NA
III	8 (38.10%)	NA	NA
sampling time (day *X* of menstruation)	10.29 ± 2.053	9.50 ± 2.212	.614
Chemotherapy regimen			
AC‐T (four cycles)	13 (61.90%)	NA	NA
TEC (six cycles)	8 (38.10%)	NA	NA
Clinical data			
SAS (score)	28.6 ± 2.8	25.4 ± 3.1	.002^b^**
SDS (score)	28.3 ± 4.48	25.4 ± 3.1	.023^a^*
E2 (pmol/L)	302.7 ± 250.6	282.2 ± 258.0	.798^b^
Hemoglobin (g/L)	120.2 ± 14.6	127.6 ± 14.1	.025^a^
Total cholesterol (mmol/L)	5.2 ± 1.1	4.8 ± 0.8	.033^b^*
Triglycerides (mmol/L)	1.7 ± 1.2	1.0 ± 0.7	.048^b^ ^*^
Cognitive performance			
MMSE (score)	30(30–29.8)	30(30–29.6)	.991^b^
NCT (s)	44.0 ± 11.8	34.8 ± 9.0	.164
LTT (s)	54.1 ± 19.7	29.9 ± 9.4	.001
SDT (s)	34.7 ± 4.5	27.2 ± 2.2	<.001
WDT (s)	8.1 ± 1.4	8.7 ± 1.2	.072
DST (s)	55.2 ± 13.2	68.5 ± 7.3	.001

*Note*: Mean ± standard deviation; median and inter‐quartile range [*M* (QU − QL)].

Abbreviations: A, doxorubicin; C, cyclophosphamide; DST, digit symbol test; E, epirubicin; LTT, line tracing test; MMSE, mini‐mental state examination; *n*, number; NCT, number connection test A; SAS, self‐rating anxiety scale; SAS, self‐rating anxiety scale; SDS, self‐rating depression scale; SDS, self‐rating depression scale; SDT, serial dotting test; T, docetaxel; WDT, auditory verbal learning memory.

^a^ *k*‐Independent samples nonparametric tests.

^b^ Two‐sample *t*‐test.

*p < .05.

**p < .01.

Paired *t*‐test showed significantly decreased E2 levels (*p* = .002), increased FSH (*p* = .001), increased LH (*p* = .001), and low fasting glucose levels (*p* = .040) between *t*_0_ and *t*_1_. In addition, the BC patients showed poorer cognitive performance in the LTT and WDT tests after chemotherapy (*p* = .002, *p* = .003, respectively). In addition, the BC patients’ showed increased anxiety at *t*_1_ compared to *t*_0_ (*p* = .033). Repeated measures ANOVA showed significant group‐by‐time interactions in triglyceride levels (*F* = 4.278, *p* = .047), E2 (*F* = 11.511, *p* = .002), FSH (*F* = 24.426, *p* < .001), and LH levels (*F* = 19.26, *p* < .001). Although BC patients had worse memory and attention after chemotherapy, there were no significant group‐by‐time interactions. In addition, although the BC group had higher cholesterol and lower hemoglobin levels compared with HC at the two time points, there were no significant between‐group differences at *t*_1_. The detailed demographics and clinical data are shown in Table 2.

**TABLE 2:** Summary of clinical characteristics and neuropsychological assessment

	Patient received chemotherapy (*n* = 21)			Healthy control (*n* = 20)			Group‐time interaction Repeated measures	
	*t*_0_	*t*_1_	Paired *t*‐test (*p*)	*t*_0_	*t*_1_	Paired *t*‐test (*p*)		
	Mean ± SD	Mean ± SD		Mean ± SD	Mean ± SD		*F*	*p*
E2 (pmol/L)	312.4 ± 56.6	116.9 ± 30.5	.002**	282.1 ± 258.0	439.1 ± 281.7	.078	11.511	.002**
FSH (IU/L)	21.5 ± 35.9	63.5 ± 43.5	.001**	24.5 ± 30.0	16.0 ± 22.3	.175	24.426	.001**
LH (IU/L)	13.3 ± 22.3	35.1 ± 23.8	.001**	14.2 ± 12.7	9.4 ± 9.0	.099	19.26	.001**
Fasting glucose (mmol/L)	5.8 ± 1.6	4.7 ± 1.3	.040*	4.8 ± 0.5	4.9 ± 0.7	.687	2.867	.100
cholesterol (mmol/L)	5.3 ± 1.22	5.02 ± 1.08	.145	4.9 ± 0.85	4.6 ± 0.7	.206	.329	.570
Triglycerides (mmol/L)	1.7 ± 1.25	2.3 ± 1.7	.070	1.0 ± 0.8	0.9 ± 0.6	.381	4.278	.047
Hemoglobin (g/L)	121.8 ± 14.9	122.3 ± 11.5	.909	131.0 ± 11.2	127.3 ± 13.1	.458	.149	.702
**Cognitive performance**								
MMSE (score)	28.2 ± 1.4	28.1 ± 1.1	.415	26.5 ± 1.5	27.1 ± 1.5	.061	.099	.754
SAS (score)	28.7 ± 2.7	31.3 ± 4.5	.033*	25.4 ± 3.5	24.5 ± 3.5	.542	3.076	.062
SDS (score)	28.7 ± 4.2	32.2 ± 5.9	.073	25.2 ± 3.0	26.9 ± 4.2	.311	.610	.442
NCT (s)	44.6 ± 11.8	41.0± 15.0	.231	34.8 ± 9.0	31.6 ± 8.7	.376	.009	.924
DST (s)	55.2 ± 13.2	55.3 ± 9.3	.946	68.5 ± 7.3	67.1 ± 6.5	.494	.820	.372
LTT (s)	54.1 ± 19.7	38.1 ± 8.7	.002**	29.9 ± 9.4	27.6 ± 9.4	.556	6.267	.018*
SDT (s)	34.7 ± 4.5	35.5 ± 7.1	.572	27.2 ± 2.2	28.6 ± 3.5	.250	.027	.870
WDT (s)	8.1 ± 1.4	6.5 ± 2.2	.003**	8.7 ± 1.2	8.1 ± 1.5	.083	9.475	.004**
Stroop‐C (s)	15.7 ± 2.8	14.4 ± 3.1	.125	11.7 ± 2.7	12.6 ± 2.5	.340	3.244	.083
Stroop‐D (s)	19.4 ± 2.7	19.2 ± 4.3	.846	13.6 ± 2.5	15.2 ± 2.0	.111	1.906	.178
Stroop‐I (s)	32.4 ± 8.3	34.0 ± 9.8	.469	20.5 ± 5.1	23.4 ± 5.5	.229	.147	.704

*Note*: Mean ± standard deviation.

Abbreviations: DST, digit symbol test; E2, estradiol; FSH, follicle stimulating hormone; LH, luteinizing hormone; LTT, line tracing test; MMSE, mini‐mental state examination; NCT, number connection test A; SAS, self‐rating anxiety scale; SD, standard deviation; SDS, self‐rating depression scale; SDT, serial dotting test; Stroop‐C, Stroop color test; Stroop‐D, Stroop word test; Stroop‐I, Stroop interference test; *t*_0_, baseline assessment; *t*_1_, after chemotherapy or at matched interval for controls; WDT, auditory verbal learning memory.

*p < .05.

**p < .01.

There were no significant differences in demographics or clinical data in the HC group between *t*_0_ and *t*_1_. The significant differences in demographics and clinical data were considered for later correlation analysis.

### 2.7 Neuroimaging results

#### 2.7.1 Baseline group comparisons

Voxel‐based independent‐sample *t*‐test revealed no significant differences (GRF correction, *p*‐value <.001 at voxel level, and *p*‐value <.05 at cluster level) in baseline hippocampus functional connectivity between the two groups (SAS and SDS scores as covariates or not).

#### 2.7.2 Within‐group comparisons

**FIGURE 1:**
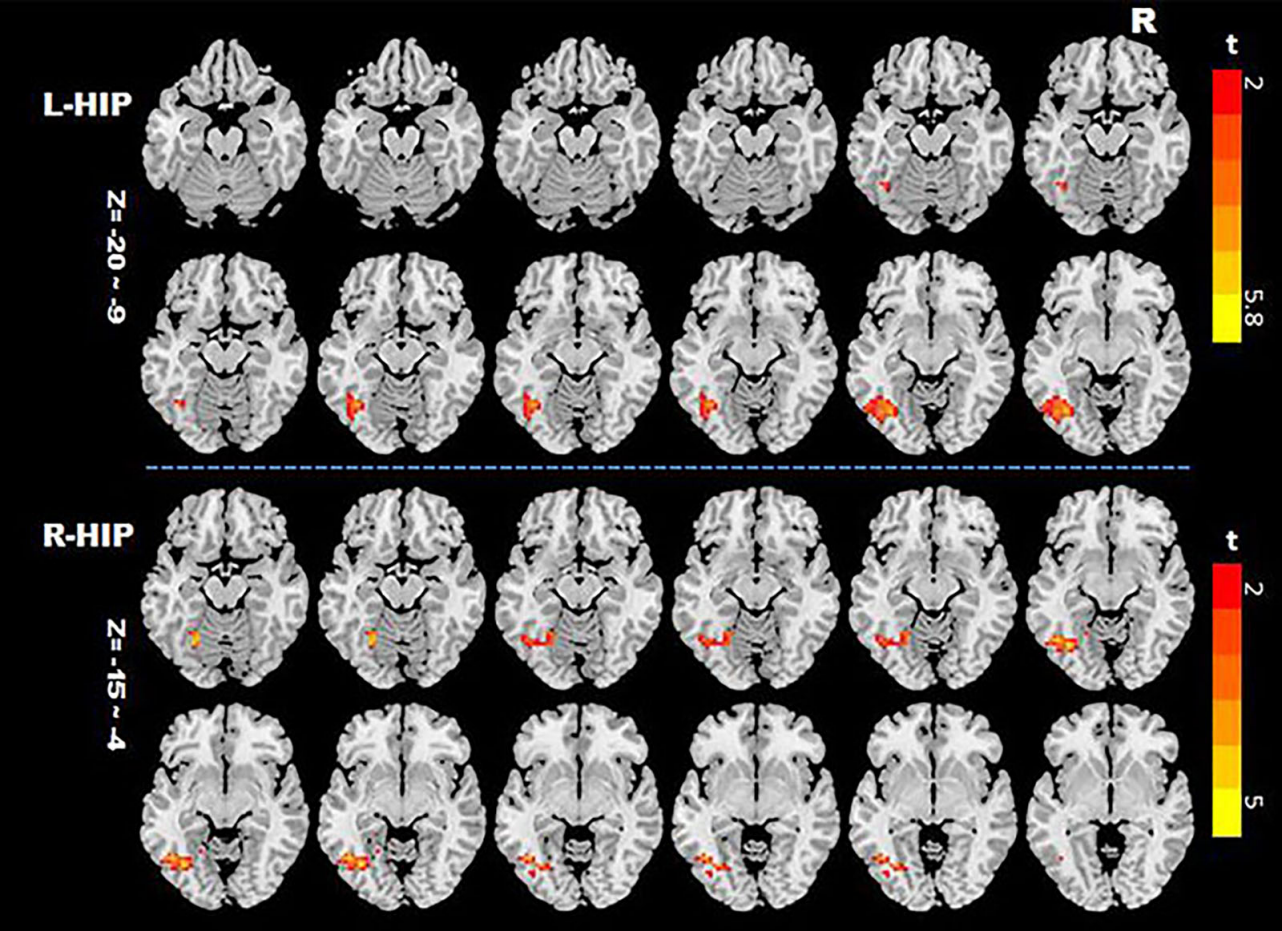
Significant within group brain functional connectivity between the hippocampus to the whole brain after chemotherapy. Voxel‐based paired *t*‐tests reveals significantly increased functional connectivity in the left hippocampus to the inferior temporal gyrus, the left fusiform gyrus, inferior occipital gyrus (Gaussian random field (GRF) correction, *p* < .001), and the right hippocampus to the left fusiform gyrus, the inferior temporal gyrus, inferior occipital gyrus, lingual gyrus, and parahippocampal gyrus after chemotherapy (GRF correction, *p* < .01). Colored regions show increasing statistical significance as labeled on the color bar (*t* map). HIP, hippocampus; L, left; R, right.

Voxel‐based paired *t*‐tests revealed significantly increased functional connectivity of the bilateral hippocampus with the left fusiform gyrus, inferior and middle temporal gyrus, inferior occipital gyrus, left lingual gyrus, and parahippocampal gyrus after chemotherapy, SAS, SDS, and gray matter as covariates (GRF correction, *p* < .001) (Table 3, Figure 1). In addition, compared with *t*_0_, the functional connectivity between the right hippocampus and the left superior frontal gyrus was decreased in the HC group at *t*_1_. After correcting for estrogen, there were no significant changes observed in hippocampus functional connectivity between *t*_0_ and *t*_1_ (GRF correction, *p* < .001).

**TABLE 3:** Statistically significant differences of brain functional connectivity in premenopausal patients after chemotherapy

	Within‐group comparisons					Group‐by‐time interaction		
	Anatomic region	No. of cluster voxel	MNI coordinates *X Y Z*	*T*‐Score (peak value)	Anatomic region	No. of cluster voxel	MNI coordinates *X Y Z*	*F*‐score (peak value)
L‐HIPPO	temporal‐Inf‐L (BA37‐L)	86	−54, −63, −6	4.984	Fusiform‐R (BA20‐R)	45	30, −3, −42	37.275
	Fusiform‐L (BA37‐L)	56	−39, −60, −9	4.924	Parahippocampal‐R (BA36‐R)	24	28, −3, −34	23.082
	Occipital‐Inf‐L	47	−43, −67, −10	5.701	temporal‐Inf‐L (BA37‐L)	36	−54, −67, −8	29.705
					Occipital‐Inf‐L	42	−36, −45, 15	27.182
					Fusiform‐L (BA37‐L)	25	−38, −59, 13	28.988
R‐HIPPO	Fusiform‐L (BA19‐L)	45	−24, −57, −15	4.751				
	temporal‐Inf‐L	35	−44, −60, −9	4.682				
	Occipital‐Inf‐L	34	−36, −66, −9	4.597				

*Note*: Gaussian random field theory correction, voxel *p*‐value <.001, cluster *p*‐value <.05. Only list the peak voxel MNI coordinates.

Abbreviations: BA, Brodmann area; HIPPO, hippocampus; Inf, inferior; L, left; MNI, Montreal Neurological Institute; R, right.

#### 2.7.3 Group‐by‐time interactions

Repeated measures analysis revealed significant group‐by‐time interactions in the left hippocampus with the bilateral fusiform gyrus, right parahippocampal gyrus, left inferior temporal gyrus, and left inferior occipital gyrus (GRF correction, *p* < .001) (Table 3) in BC patients (SAS, SDS, and gray matter as covariates). Most of these interaction brain regions were overlapped with the brain areas survived in with‐group comparisons.

#### 2.7.4 Correlation analysis

**FIGURE 2:**
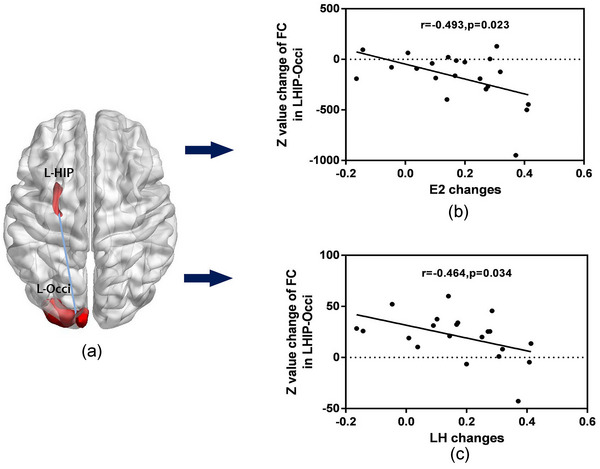
**A significant group‐by‐time interactions in left inferior occipital gyrus and correlation analysis of functional connectivity changes with clinical variables changes in patients**. A significant group‐by‐time interaction of functional connectivity was observed in the left hippocampal to the left inferior occipital gyrus in premenopausal patients (a), whereas the healthy volunteers did not. Functional connectivity in these regions negatively correlated with the E2 (b) and luteinizing hormone (LH) changes (c) (all *p* < .05). HIP, hippocampus; L, left; Occi, inferior occipital gyrus.

Correlation analysis was conducted between the significant functional connectivity changes and changes in clinical data between *t*_0_ and *t*_1_. Pearson correlation analysis showed significant negative correlation between the functional connectivity changes in left hippocampus to the left inferior occipital gyrus and changes in E2 (*r* = −.493, *p* = .023) and in LH (*r* = −.464, *p* = .034), corrected for multiple comparisons respectively (Figure 2). Furthermore, the pretreatment SDT was negatively correlated with lower HB levels (*r* = −.463, *p* = .034) in the pretreatment period. No other correlations were observed between hippocampal connectivity changes and neuropsychological and blood biochemical changes. There were no significant correlations observed in the HC.

## 3 DISCUSSION

This study showed increased functional connectivity of the bilateral hippocampus with the posterior region of brain after chemotherapy in CIA women. Altered functional connectivity between the left hippocampus with the left inferior occipital gyrus was significant correlated with E2 and LH changes. In addition, CIA patients mainly had cognitive dysfunction in memory and visual mobility. The results suggest that chemotherapy may affect hippocampal‐prefrontal cortical circuit which mediates visual processing in premenopausal patients, E2 may be involved in this process.

These findings were partially consistent with previous studies examining the cognitive effects and brain activation changes of CRCI (Apple et al., 2017; Cheng et al., 2017; Conroy et al., 2013; Perrier et al., 2020; Stouten‐Kemperman et al., 2015). In this study, hippocampal–cortical functional connectivity changes were mainly observed between the left hippocampus and the left temporo–occipital areas after treatment in the CIA group. The temporal lobe was one of the most affected regions in the early stages of chemotherapy (Cheng et al., 2017; Lepage et al 2014., Feng et al., 2020; Stouten‐Kemperman et al., 2015), consistent with previous studies (Deprez et al., 2012; Kesler et al., 2015; McDonald et al., 2010). In addition, significantly increased left‐sided hippocampal functional connectivity was present between the left fusiform gyrus and the left inferior occipital gyrus. The fusiform gyrus known as the lateral occipital gyrus is part of the temporal and occipital lobes. It is involved in face recognition tasks, memory task, and vision‐related neural activities (Li et al., 2021; Loffler et al., 2005; Silverstein et al., 2010). The inferior temporal gyrus participates in high‐level visual processing, also involved in complex object features (such as overall shape), facial perception, and number recognition (Kravitz et al., 2013). The occipital lobe contains the primary visual cortex, which is involved in visual information processing (Rosenke et al., 2021). The inferior temporal gyrus, the fusiform gyrus, the lateral occipital lobe, and the hippocampal gyrus work together to process visual images and perceive objects. These processes may contribute to recognition and memory. The present study showed abnormal functional connectivity in these brain areas. These findings provide evidence for chemotherapy‐induced aberrant neural circuits which support visual memory and recognition processing. Cognitive dysfunctions in CIA patients may be closely correlated with abnormal functional connectivity in the hippocampus and temporo–occipital areas.

The CIA patients showed poorer visual processing in neuropsychological tests. Previous prospective studies showed cognitive impairment in multiple domains involving attention, memory, psychomotor, executive functioning, information processing speed, and damage of the visuospatial domains (Luine & Frankfurt, 2020; Peukert et al., 2020). Findings of the present study are partly consistent with previous CRCI related studies. Our previous study reported visual, auditory memory, and executive function impairment in chemotherapy‐treated BC patients (Feng et al., 2020). Further, the study showed overlapping cognitive impairments in CIA patients. It was unclear why other cognitive functioning domains such as executive functioning were not observed in the CIA patients. We hypothesized that this could be attributed to the fact that the present study only included premenopausal patients, whereas previous studies included both postmenopausal and premenopausal patients. Specific cognitive impairment in chemotherapy treated patients seems relevant for pretreatment menopausal status consistent with findings reported by Conroy et al. (2013). They reported that premenopausal patients who underwent CIA showed increased magnitudes of activation or deactivation during the cognitive tasks compared with postmenopausal BC patients, suggesting the pattern of change in brain activity according to pretreatment menopausal status. However, they reported some different brain areas during the cognitive tasks. The inconsistences partially account for different MRI analyses, our analyses here were resting state while Conroy et al. were task‐based, other factors such as different visual function tests and the duration of the follow‐up period may also have influenced the results. Further studies should be conducted to determine whether menstrual status before treatment affects cognitive impairment after chemotherapy.

In this study, E2 were negatively associated with functional connectivity changes between the left hippocampus and the left inferior occipital gyrus. The hippocampal connectivity changes were thought to be a result of the drop in estrogen levels due to chemotherapy‐induced suppression of ovarian function. It is hypothesised that women have stronger automatic visual processing with increased estrogen and/or progesterone levels (Farage et al., 2008; Leeners et al., 2017; Poromaa & Gingnell, 2014; Sherwin, 2012). In the present study, the CIA patients had decreased estrogen levels which could have led to the decreased visuospatial ability (Mordecai et al., 2008; Wharton et al., 2008; Zilles et al., 2016). The increased functional connectivity between the hippocampus and the left inferior occipital gyrus might be related to compensatory mechanisms for visuospatial deficits in the CIA patients to maintain cognitive function, acting to enhance their functional connectivity. Gonadotropin hormone releasing hormone agonists (GnRHa) and surgical menopause provided a powerful model to study the neural effects of abrupt decrease in estrogen hormone in premenopausal women (Sherwin & Phillips, 2006; Vearncombe et al., 2009; Wharton et al., 2008). These studies generally show that decreased estrogen is associated with reduce activation in several brain regions that are important to the visual working memory, such as precuneus, posterior cingulate, paracentral lobule, parahippocampus, and middle temporal gyrus. In addition, the visual function area may differ between tasks. Results of the present study showed some similar functional areas, such as inferior occipital gyrus, parahippocampus, and temporal gyrus in CIA patients, suggesting that this region may be particularly sensitive to abrupt decrease in estrogen levels.

In this study, CIA patients showed increased LH and FSH levels. Higher circulating levels of LH and FSH were attributed to decreased negative feedback on estrogen release. Some studies report that high levels of LH and FSH were associated with depression (Gu et al., 2018; Soares, 2016). According to Freeman (2010), depressive symptoms were significantly associated with greater variability in FSH and estradiol levels. They reported that women with a history of depression had a high risk of depressive symptoms upon transitioning to menopause. We speculate that CIA related drastic hormone changes will adversely affect patients’ emotions. Previous studies have reported that excessive negative emotional burden before treatment might affects brain activation and cognition (Jung et al., 2017; Reinecke et al., 2014). However, in the study, there was no direct evidence linking sex hormones changes to increased negative mood after chemotherapy. This may be attributed to the fact that CIA patients are young women with high baseline cognitive reserve and brain compensatory ability.

Interestingly, we found that a low pretreatment hemoglobin level was correlated to the depression scores. Evidence from the literature supports the influence of metabolism on limbic system function, mood regulation, and emotional regulation disorder in the context of metabolic dysfunction (Araújo et al., 2002; Berent‐Spillson et al., 2017; Loo et al., 2013). Vearncombe et al. (2009) reported that low pretreatment hemoglobin levels could indicate cognitive impairment after chemotherapy. This study did not find any association between post‐chemotherapy hemoglobin levels and cognitive impairment, which may be related to the use of hematopoietic drugs. In addition, BC patients showed increased cholesterol, triglycerides, and fasting blood glucose levels after chemotherapy. High pretreatment triglyceride and total cholesterol levels may be related to the BC hormone receptor type (Kim et al., 2009), and post‐chemotherapy dyslipidemia may be related to CIA induced drastic hormone fluctuations (Delgobo et al., 2019; Tian et al., 2019). Hyperglycemia, hypercholesterolemia, and triglycerides are risk factors for vascular disease associated with cognitive dysfunction. Both neuropsychiatric diseases and AD had been associated with lipid metabolic changes of the frontal cortex in humans as well as in animal models (Bories et al., 2017). However, it remain unclear whether abnormal lipid metabolism affects cognitive functions by influencing the metabolic in the cerebral cortex or building up into vascular disease leading to cognitive dysfunction. Although there is no direct evidence linking metabolism to cognitive dysfunction in the study, we believe that chemotherapy‐related cognitive dysfunction may be a cumulative effect, including effects of cancer itself and chemotherapy (Cheung et al., 2015), which overlap with those thought to be involved in the deleterious effects of dramatic hormonal changes and persistent abnormal lipid metabolism, suggesting possible compounding effects.

This study had some limitations. First, the female ovarian hormones fluctuate during menstrual cycle. In addition, the hormonal levels may vary widely among individuals. However, due to the operation time and chemotherapy schedule, we could not guarantee that every blood sample would be collected during the same days of menstruation. Moreover, some patients had CIA and blood samples were collected randomly in the CIA group. Second, we did not control for use of lipid‐lowering drugs and hypoglycemic agents in the study. Therefore, this limits the assessment of cognitive impairment due to metabolic abnormalities. Third, this study did not have a CIA‐negative control group. Therefore, it was difficult to dissociate the effects of CIA and chemicals on cognitive functions. However, it would be difficult to achieve an adequate sample size in the CIA‐negative control group in clinical practice. Finally, this study only evaluated the general cognitive ability; therefore, chemotherapy‐related specific visuospatial cognitive impairment could not be evaluated.

## 4 CONCLUSION

In summary, this study showed that chemotherapeutic agents were more likely to affect the brain network involved in visual processing and memory in premenopausal women. In addition, the abrupt decrease of estrogen levels may be involved in the regulation of visual processing. Direct neurotoxicity and chemotherapy‐induced abrupt hormonal changes suggest that CIA may increase the harmful effects of CRCI. The regulation of neuroplasticity by E2 provides a framework for predicting estrogen‐dependent cognitive function in the brain and can be used as a potential biomarker. Although the effects of estrogen changes on the human brain are still complicated and controversial, the cognitive effects of immediate and long‐term decreased hormone levels remain unknown. Therefore, further longitudinal studies should be conducted.

## AUTHOR CONTRIBUTIONS

Yingying Zhuang conducted the subject's MRI examination and participated in the interpretation and analysis of data and drafted the manuscript. Lili Guo participated in the interpretation of the data and the revision of the manuscript. Wei Huang participated in the MRI examination and the collection of clinical data. Genji Bo recruited chemotherapy patients and normal control population. Jiandong Zhang and Zhaohuan Zhu contributed to the statistical analysis. Yun Feng conceived of the study, participated in its design, and was responsible for the preparation of the manuscript. The authors read and approved the final manuscript.

## CONFLICT OF INTEREST STATEMENT

The authors declare that they have no conflict of interest.

### PEER REVIEW

The peer review history for this article is available at https://publons.com/publon/10.1002/brb3.3039.

## Data Availability

All data generated or analyzed during this study are included in this article.
